# Suicidal crisis: A common cause of hospitalisation in adolescents. introducing an innovative program

**DOI:** 10.1192/j.eurpsy.2023.287

**Published:** 2023-07-19

**Authors:** A. Vargas Castro

**Affiliations:** 1 Child & Adolescent Psychiatry, Hôpital de la Pitié Salpêtrière, Paris; 2 Child & Adolescent Psychiatry, Centre Hospitalier de Versailles, Versailles, France

## Abstract

**Introduction:**

Attempts at suicide and suicidal tendencies have been the most frequent and common reasons for adolescent inpatient treatments since the last Covid-19 pandemic. Indeed, the WHO has reported that the second most frequent cause of mortality in adolescents is related to suicidal acts.

**Objectives:**

The Sun Project, which is a pilot research program aimed at finding a comprehensive set of steps for treatment, has been developed at the Versailles Medical Center in France and provides multidisciplinary tools to tackle this phenomenon.

**Methods:**

This retrospective observational research with a cohort of fifty people between pre-teen and adolescence has taken advantage of different elements of specific psychotherapeutical approaches such as Acceptance and Commitment Therapy, Interpersonal Psychotherapy, Compassion, Narrative, Dialectical and Cognitive Behavioural Therapies in relation to Family-Based Therapy and employs elements of Emotional Freedom Techniques as well as the CESAR program (Cognitive, Emotional and psycho-Social Avatar Reinforcement program).

**Results:**

The results have been very positive and this is attributed to the transdisciplinary network around each patient, family inclusion and the multi-pronged psychotherapeutic approach based on functional analysis of every patient’s situation.

**Conclusions:**

In short, The Sun Project has shown that these approaches and interventions give excellent and rapid outcomes in pre-teens and adolescents suffering from suicide related thoughts and acts.

**Disclosure of Interest:**

None Declared

